# H_2_S modulates *BrSDH1-1* alternative splicing to induce stomatal closure in Chinese cabbage

**DOI:** 10.1093/hr/uhaf214

**Published:** 2025-08-22

**Authors:** Jiao Zhang, Liping Zhang, Xiaoli Han, Guoxiang Wang, Jiaqi Sun, Danmei Liu, Zhiqiang Liu, Yanxi Pei, Zhuping Jin

**Affiliations:** School of Life Science, Shanxi University, Taiyuan 030031, China; Shanxi Key Laboratory for Research and Development of Regional Plants, Taiyuan 030031, China; School of Life Science, Shanxi University, Taiyuan 030031, China; Shanxi Key Laboratory for Research and Development of Regional Plants, Taiyuan 030031, China; School of Life Science, Shanxi University, Taiyuan 030031, China; Shanxi Key Laboratory for Research and Development of Regional Plants, Taiyuan 030031, China; School of Life Science, Shanxi University, Taiyuan 030031, China; Shanxi Key Laboratory for Research and Development of Regional Plants, Taiyuan 030031, China; School of Life Science, Shanxi University, Taiyuan 030031, China; Shanxi Key Laboratory for Research and Development of Regional Plants, Taiyuan 030031, China; School of Life Science, Shanxi University, Taiyuan 030031, China; Shanxi Key Laboratory for Research and Development of Regional Plants, Taiyuan 030031, China; School of Life Science, Shanxi University, Taiyuan 030031, China; Shanxi Key Laboratory for Research and Development of Regional Plants, Taiyuan 030031, China; School of Life Science, Shanxi University, Taiyuan 030031, China; Shanxi Key Laboratory for Research and Development of Regional Plants, Taiyuan 030031, China; School of Life Science, Shanxi University, Taiyuan 030031, China; Shanxi Key Laboratory for Research and Development of Regional Plants, Taiyuan 030031, China

## Abstract

Hydrogen sulfide (H_2_S), a gasotransmitter molecule, plays critical roles in stomatal closure and cellular bioenergetics. Alternative splicing (AS) is a key regulatory mechanism during plant development and stress responses; however, the interplay between H_2_S signaling and AS in drought tolerance remains unexplored in Chinese cabbage. In this study, we found that the mitochondrial inner membrane enzyme succinate dehydrogenase (SDH) responds to H_2_S signaling during stomatal closure. Silencing of *BrSDH1-1* impaired the effects of H_2_S on stomatal closure, SDH activity, and ATP production. RNA-Seq analysis revealed that H_2_S modulates the AS of *BrSDH1-1*, resulting in transcript variants with differential expression. Overexpression of *BrSDH1-1A* and *BrSDH1-1C* in *Arabidopsis* enhanced drought resistance, whereas *BrSDH1-1B* had no significant effect. H_2_S enhanced SDH activity and ATP production, promoted stomatal closure, and reduced excess reactive oxygen species (ROS) in OE-*BrSDH1-1A* and OE-*BrSDH1-1*C lines but not in OE-*BrSDH1-1B*. Furthermore, biotin-switch assays demonstrated that H_2_S induced persulfidation of BrSDH1-1A and BrSDH1-1C, with no effect on variant BrSDH1-1B. These findings reveal a novel regulatory mechanism by which H_2_S modulates *BrSDH1-1* splicing to mediate stomatal closure and improve drought tolerance, offering valuable molecular insights for enhancing stress resilience in horticultural crops.

## Introduction

Chinese cabbage (*Brassica rapa*) originates from China and possesses an extremely rich genetic diversity. It is a major vegetable crop in China, Korea, Japan, and Southeast Asia. *Brassica rapa* requires substantial water during cultivation, and drought is the primary environmental factor limiting its growth and development [1]. Whole-genome analysis and the improved database (BRAD, http://brassicadb.cn) provide strong support for molecular biology research on *B. rapa* [2, 3]. It also serves as a bridge for studying cultivated crops by utilizing the rich genomic information of *Arabidopsis thaliana*. There is clear collinearity between the *B. rapa* and *A. thaliana* genomes. Most researchers believe the *B. rapa* genome results from gene duplication caused by triplication of the diploid ancestral genome or whole-genome polyploidization, with multiple gene copies potentially contributing to phenotypic diversity *via* functional divergence. However, the extent of repeated regions in this collinearity remains unclear [2]. The polyploidy of the *B. rapa* genome makes functional studies highly challenging. Genes in *A. thaliana* often correspond to multiple homologs in the *B. rapa* genome, significantly increasing the workload for functional analysis. Alternative splicing (AS) widely occurs during genome evolution, enhancing transcriptome diversity and plasticity. This modulates gene expression and improves responsiveness to environmental stress [4]. Whether these homologous multicopy genes are merely redundant or have diverged functionally remains unresolved due to limited experimental evidence, making it a key and compelling area of research.

With global climate change, such as global warming, drought has become one of the main environmental stressors that severely limit plant growth and development. As a key vegetable crop, Chinese cabbage holds significant economic importance. However, its leafy nature makes it highly vulnerable to drought stress, which always arrest growth and endanger yield stability [5]. Current approaches to enhancing drought resistance primarily focus on internal structural adaptations and external morphological adjustments, such as alterations in cuticle thickness, stomatal characteristics, and root architecture. Additionally, key gene pathways involved in stress perception, signal transduction, and the activation of stress-responsive genes, along with the interplay between exogenous hormones and plants, also contribute to drought stress tolerance in vegetables [6, 7]. Stomata are formed by pairs of kidney-shaped guard cells (GCs), specialized epidermal cells on the leaf surface that balance carbon dioxide (CO_2_) uptake for photosynthesis and water loss to optimize responses to various environmental stimuli [8]. GCs perceive diverse signals and modulate stomatal aperture by adjusting turgor pressure through coordinated ion and water transport across plasma and vacuolar membranes, as well as cytosolic metabolite dynamics. Inorganic ions are crucial for stomatal opening early in the day, whereas sucrose plays a key role in maintaining stomatal opening later in the circadian rhythm [9]. The external stimuli activate cellular receptors or sensors, triggering signaling pathways involving phytohormones, redox status, and Ca^2+^ dynamics, which induce physiological changes and ultimately drive stomatal movement [10]. Accurately regulating stomatal movement remains a challenging scientific problem that garners significant attention and holds great practical importance for crop production in arid regions.

Research over recent decades confirms that stomatal movement requires ATP, yet the source of GCs ATP remains controversial. Early studies have found that exogenous ATP can be absorbed by the leaf epidermis, significantly increasing the stomatal diameter [11]. Subsequently, from the perspective of stomatal dynamics, synthetic light-gated K^+^ channels expressed 2.2 times in *Arabidopsis* stomatal GCs significantly increased stomatal opening under illumination and the speed of stomatal closure after irradiation, thereby enhancing plant biomass [12]. Another report suggests that glucose is the main energy source for stomatal movement and describes the molecular mechanism by which light regulated the opening and closing of GCs through membrane ion transport and starch metabolism [13]. Recent evidence confirms that GCs exhibit low photosynthetic capacity, relying predominantly on mitochondrial oxidative phosphorylation for ATP [14]. However, how the GCs membrane precisely controls stomatal movement *via* ion transport and energy metabolism remains unclear.

The primary source of energy in eukaryotic cells is mitochondria. A series of complexes (I, II, III, IV, V) located on the inner mitochondrial membrane form a respiratory chain, which transports electrons and H^+^, utilizes O_2_, and ultimately produces ATP and H_2_O. Complex II, also known as succinate dehydrogenase (SDH), catalyzes the oxidation of succinic acid to fumarate and the reduction of ubiquinone to panthenol. It serves as a hub connecting the tricarboxylic acid cycle and electron transport chain (ETC), playing a central role in mitochondrial metabolism [15]. The structure of SDH is shown in Fig. S1, and the gene IDs of *A. thaliana* and *B. rapa* are listed in Table S1. *Arabidopsis* SDH clearly contains two outer membrane proteins (flavin SDH1 and ferrothionein SDH2), two small complete membrane proteins (SDH3 and SDH4), and four additional subunits (SDH5–SDH8); though SDH5–SDH8 do not exhibit clear functional domains in sequence analysis [16]. The SDH1 subunit is encoded by nuclear genes *SDH1-1* and *SDH1-2*. *SDH1-1* is highly expressed in GCs, whereas *SDH1-2* has low overall expression, and its mutation does not affect plant growth. Thus, SDH1-1 is essential for plant gametophyte development, with homozygous mutations being lethal [15]. Decreased SDH activity in heterozygous mutant *SDH1-1/sdh1-1* results in increased stomatal conductance and promotes photosynthesis and nitrogen assimilation [17]. Simultaneously, point mutation at the succinate binding site of SDH1-1 produced the mutant *dsr1* (*disrupted stress response 1*), which exhibited reduced SDH activity and increased sensitivity to specific bacteria [18]. Correspondingly, the exogenous application of thenoyl trifluoroacetone (TTFA), an SDH inhibitor, significantly induced reactive oxygen species (ROS) production, thereby inhibiting the growth and development of *A. thaliana* and rice [19]. In conclusion, SDH1-1 is the only protein encoding functional flavins; SDH1-1 plays a more direct and prominent role in regulating plant growth and metabolism. Therefore, the function of BrSDH1-1 should be investigated; however, this is challenging due to the presence of duplicate homologs in *B. rapa* compared to *A. thaliana*.

In recent years, increasing evidence has shown that hydrogen sulfide (H_2_S) plays a significant physiological role as a gasotransmitter in plants. The specific physiological functions of H_2_S in plants are evident throughout the entire growth and development process, including promoting seed germination, facilitating root morphogenesis, enhancing leaf photosynthesis, inducing stomatal closure, delaying senescence, and more [20]. Exogenous application of H_2_S at physiological concentrations also helps plants resist abiotic and biotic stresses. Substantial evidence demonstrates that H_2_S signaling induces stomatal closure *via* abscisic acid (ABA)- and H_2_O_2_-dependent pathways while synergizing with nitric oxide (NO) and phytohormone networks. This regulation primarily targets key GCs ion channels and signaling hubs to modulate stomatal dynamics [21].

Most functional studies of H_2_S focus only on physiological or molecular changes, rarely identifying the actual targets of H_2_S action. In recent years, breakthroughs have been made in understanding the signaling mechanisms of H_2_S molecules. A key mechanism is the mediation of posttranslational covalent modification of protein cysteine residues, converting thiol groups (-SH), sulfur hydroxyl groups (-S-OH), etc., into -S-SH, known as sulfhydration modification (S-sulfhydration or persulfidation, denoted as -S-SH modification), which leads to changes in protein function, subcellular localization, stability, etc. This signaling mechanism of H_2_S is also a major reason for its broad and diverse physiological functions [22, 23]. Analytical methods for detecting plant protein persulfidation modifications, based on biotin switch assay, liquid chromatography, mass spectrometry, and other techniques, have also been developed [24]. This persulfidation modification has become a hot topic in the study of H_2_S signaling mechanisms.

Research on the molecular targets and mechanisms of H_2_S in plant cells has also achieved many breakthroughs. It was found that -S-SH modification induced by ABA promotes the production of DES and facilitates stomatal closure [25]. Further studies revealed that H_2_S mediates the -S-SH modification of Cys250 in the positive regulatory factor ABI4 within the ABA signaling pathway. This posttranslational modification enhances the transcriptional activation of the downstream target MAPKKK18 of ABI4, and the H_2_S signal regulates the plant ABA response through this pathway [26]. H_2_S-mediated -S-SH modification can also alter the conformation of the key kinase protein SnRK2.6 in the ABA signaling pathway, thereby increasing the efficiency of ATP-γ-phosphate group transfer by SnRK2.6 and enhancing its kinase activity [27]. This -S-SH modification can also interfere with actin polymerization, thereby affecting the development of plant root hairs [28]. Recent studies by our research group have shown that H_2_S signals respond to drought stress by balancing stomatal and non-stomatal factors through the persulfidation of the target protein RuBisCO in Chinese cabbage [29], regulating energy metabolism by persulfidating SDH to induce stomatal closure [30], and controlling plant flowering by persulfidating Flowering Locus C (FLC) in Chinese cabbage and the splicing factor U2AF65a [31, 32]. These results implied that H_2_S plays multiple roles in development and stress response by regulating the bioenergetic metabolism, with AS potentially serving as a novel mechanism of its action. However, the exploration of the physiological function and molecular mechanism of H_2_S signaling in Chinese cabbage from the perspective of energy metabolism, such as BrSDH1-1, remains lacking.

**Figure 3:**
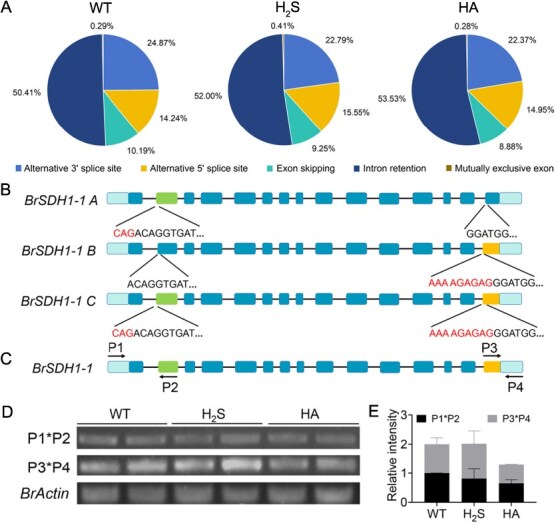
Summary of AS events in *B. rapa*. (A) Distribution of different modes of AS events with the treatment of H_2_S and HA. (B) Gene models of each *BrSDH1-1* isoform are displayed. Squares indicate the UTRs and exons, with the AS sites and their sequences annotated. (C) The specific primers are designed to distinguish the transcript of *BrSDH1-1*. Amplification using primers P1 and P2 yields *BrSDH1-1A* and *BrSDH1-1C*, and amplification by P3 and P4 yields *BrSDH1-1B* and *BrSDH1-1C*. (D, E) RT-PCR analysis of *BrSDH1-1A*, *BrSDH1-1B*, and *BrSDH1-1C* in WT with the treatment of H_2_S and HA. *BrACTIN* is an internal control. ‘P1*P2’ indicates the abundance sum of *BrSDH1-1A* and *BrSDH1-1C*; ‘P3*P4’ indicates the abundance sum of *BrSDH1-1B* and *BrSDH1-1C.*

In this study, we demonstrated that H_2_S-induced stomatal closure is partially dependent on BrSDH1-1 by regulating energy metabolism in Chinese cabbage. RNA-Seq revealed that H_2_S regulates the AS of *BrSDH1-1* to produce three transcripts that respond differently to H_2_S in stomatal closure, ROS equilibrium, and bioenergetic synthesis. Moreover, the molecular mechanism of H_2_S was elucidated at both the posttranscriptional level and in the posttranslational modification of proteins, strengthening the foundation for its broader application in crop breeding.

## Results

### H_2_S induced stomatal closure through BrSDH1-1 partially

**Figure 1:**
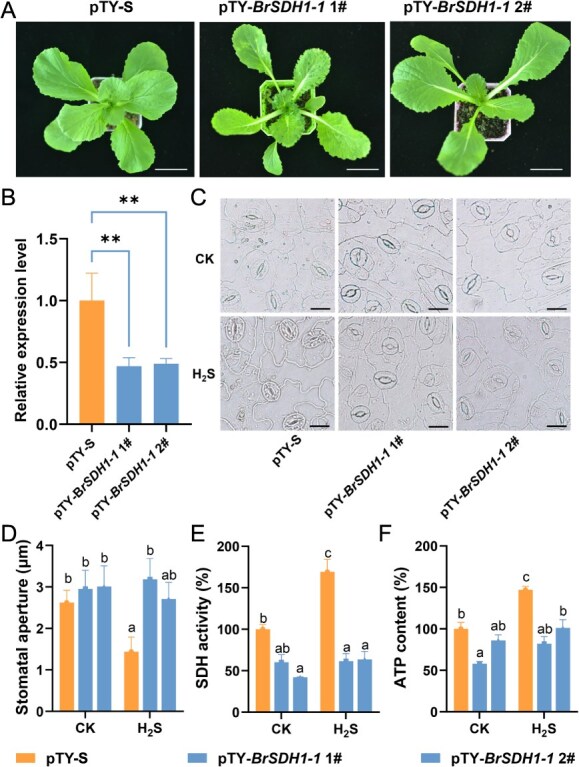
Function analysis of *BrSDH1-1* in *B. rapa*. (A) Phenotype of *BrSDH1-1*-silenced and control plants. Bar = 4 cm. (B) Silent efficiency identification of *BrSDH1-1* between pTY-S plants and pTY-*BrSDH1-1* plants by qPCR. *BrACTIN* worked as an internal reference. One-way ANOVA for detecting significant differences (**P < 0.01). (C, D) Stomatal aperture of pTY-S plants and pTY-*BrSDH1-1* plants were observed with the fumigation of H_2_S. (E) SDH activity and (F) ATP content was measured in pTY-S plants and pTY-*BrSDH1-1* plants with the fumigation of H_2_S. For D–F, two-way ANOVA determined the significant differences (*P* < 0.05).

Our previous study revealed that H_2_S activates SDH to induce stomatal closure in *A. thaliana* [30]; however, the mechanism by which H_2_S affects BrSDH1-1 in Chinese cabbage remains unclear. Sequence alignment showed that *BrSDH1-1* has two homologous genes in Chinese cabbage compared to *A. thaliana*, with gene IDs *BraA07g017190.3C* and *BraA09g009390.3C*, respectively (Fig. S3; Brara_Chiifu_V3.0, available at http://www.brassicadb.cn). The high sequence similarity between *BraA07g017190.3C* and *BraA09g009390.3C* allows for co-silencing to study the function of *BrSDH1-1*. Two silenced lines, pTY-*BrSDH1-1* 1# and pTY-*BrSDH1-1* 2#, were generated using virus-induced gene silencing (VIGS; Fig. S2). As shown in Fig. 1A, *BrSDH1-1-*silenced plants exhibited significantly weaker growth, smaller plant size, and yellowing of leaves compared to the control plants (pTY-S). The expression level of *BrSDH1-1* in the pTY-*BrSDH1-1* lines was markedly reduced compared to pTY-S plants, indicating effective gene silencing (Fig. 1B). The silenced lines displayed larger stomatal apertures than the control plants, suggesting that BrSDH1-1 influences stomatal movement (Fig. 1C and D). Then the role of H_2_S in BrSDH1-1-related stomatal closure was examined. It was evident that pTY*-BrSDH1-1* 1# showed no response to H_2_S, while pTY*-BrSDH1-1* 2# exhibited a slightly reduced stomatal aperture, though significantly less than that observed in the pTY-S plants (Fig. 1C and D). This indicated that H_2_S-mediated stomatal closure is partially dependent on BrSDH1-1. As SDH1-1 is a key component of the mitochondrial ETC, both SDH activity and ATP content were reduced in *BrSDH1-1*-silenced plants (Fig. 1E and F). The results revealed that H_2_S significantly enhanced SDH activity and promoted ATP production in the pTY-S plants, but these effects were diminished in the pTY-*BrSDH1-1* plants (Fig. 1E and F). These findings demonstrated that H_2_S mediates stomatal closure and energy metabolism through BrSDH1-1, and the underlying mechanism warrants further investigation.

### H_2_S had no effect on the expression level of *BrSDH1-1*

**Figure 2:**
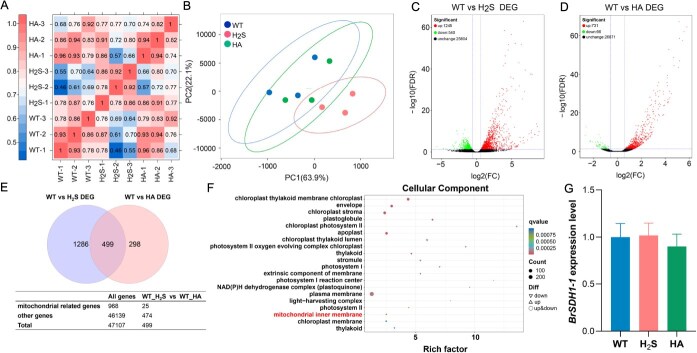
Data analysis of RNA-Seq in *B. rapa* with the treatment of H_2_S and HA. (A) Correlation analysis between different samples. (B) PCA plot of transcriptome profiles from different conditions. (C, D) Volcano plots analysis of the DEGs in H_2_S and HA groups. (E) Venn diagrams showed the number of DEGs in H_2_S and HA groups and the number of mitochondrial-related genes was displayed in the table. (F) GO enrichment analysis of DEGs (cellular component). (G) Effect of H_2_S and HA on the expression level of *BrSDH1-1*.

Since H_2_S has a significant impact on plant growth, development, and stress response, we performed full-length RNA sequencing on 10-day-old Chinese cabbage seedlings to analyze the mechanism of H_2_S on *BrSDH1-1*. A total of 23.05 GB of clean data were obtained after filtering low-quality reads and adapter sequences using the nanopore-sequencing platform (Table S3). Pearson correlation analysis of H_2_S and HA treatments revealed a strong correlation among the samples (Fig. 2A). Principal component analysis (PCA) showed that exogenous H_2_S treatment clustered separately from both the WT and HA groups (Fig. 2B), suggesting that Chinese cabbage may exhibit a notable response to H_2_S signaling. We used FDR = 0.05 and FC = 1.5 as thresholds to screen for DEGs. A total of 2584 DEGs were identified in the H_2_S and HA groups. Notably, the number of DEGs induced by H_2_S was approximately twice that induced by HA. Volcano plot analysis revealed that over 70% of genes were upregulated in both H_2_S and HA treatments (Fig. 2C and D). A Venn diagram illustrated the distribution of DEGs, identifying 499 genes that were commonly differentially expressed in both groups (Fig. 2E). To further characterize the key proteins regulated by H_2_S, we performed GO enrichment analysis on the DEGs under different treatments. Interestingly, within the cellular component category, only genes associated with the mitochondrial inner membrane showed upregulated expression in response to H_2_S (Fig. 2F). However, BrSDH1-1, an important component of the mitochondrial inner membrane, was not significantly regulated by H_2_S (Table S4). Subsequent experimental data also confirmed that *BrSDH1-1* expression was not induced by either H_2_S or HA (Fig. 2G). This observation may be related to the complex and diverse regulatory mechanisms associated with H_2_S.

### H_2_S modulated the AS of *BrSDH1-1*

Recent research indicated that H_2_S not only regulates differential gene expression but also affects AS at the posttranscriptional level. To further investigate the role of H_2_S, we analyzed transcriptomic data specifically from the perspective of AS. Consistent with observations in *A. thaliana*, intron retention was the predominant category of AS events in Chinese cabbage, accounting for more than 50% (Fig. 3A). However, the proportion of specific AS types shifted following exogenous application of H_2_S or HA, suggesting that both compounds can induce widespread AS changes (Fig. 3A). Further analysis of H_2_S influence on *BrSDH1-1* splicing patterns revealed that AS was induced in *BraA07g017190.3C* but not in *BraA09g009390.3C*. Therefore, we concentrated on the functional impact of H_2_S on *BraA07g017190.3C*. Exogenous application of H_2_S and HA triggered an A3SS event in both the first and penultimate exons of *BraA07g017190.3C*, resulting in a portion of the intron being incorporated into the coding sequence. Consequently, the original transcript was designated *BrSDH1-1A*, while the two novel transcripts were termed *BrSDH1-1B* and *BrSDH1-1*C, respectively (Fig. 3B). Specific primers were designed at two splice junctions to quantify the abundance of *BrSDH1-1A*, *BrSDH1-1B*, and *BrSDH1-1*C (Fig. 3C). Experimental results showed that H_2_S treatment upregulated *BrSDH1-1B* and *BrSDH1-1*C, while downregulating *BrSDH1-1A* and *BrSDH1-1*C. Taken together, these findings indicated that H_2_S specifically promoted the expression of *BrSDH1-1B* (Fig. 3D and E). In contrast, exogenous HA suppressed the expression of all *BrSDH1-1* variants (Fig. 3D and E). These observations suggest that H_2_S may contribute to cellular homeostasis by modulating the expression of *BrSDH1-1A*, *BrSDH1-1B*, and *BrSDH1-1C*. As numerous studies have demonstrated that AS-derived transcripts may undergo functional divergence, it is therefore essential to investigate the specific roles of *BrSDH1-1* splice variants.

### Splicing transcripts of *BrSDH1-1* performed different expression abundance

**Figure 4:**
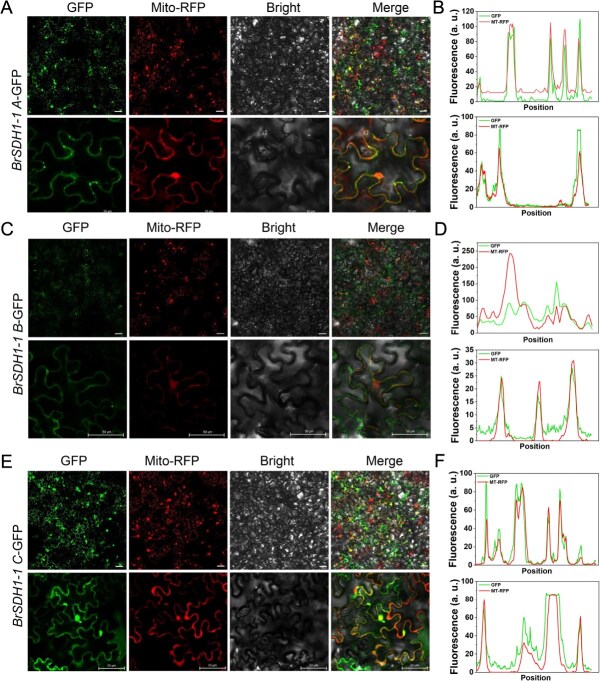
Characterization abundance of different transcripts of BrSDH1-1 quantitatively in tobacco leaves. Subcellular localization of (A) BrSDH1-1A, (C) BrSDH1-1B, and (E) BrSDH1-1C. Bar = 50 μm. (B, D, and F) The distributions and intensities analysis of GFP and RFP signal indicated in (A), (C), and (E). MT-RFP, mitochondria. a.u., arbitrary units.

We first used quantitative injection in tobacco leaves to observe the subcellular localization characteristics of transcripts BrSDH1-1A, BrSDH1-1B, and BrSDH1-1C (Fig. S4A). The results showed that all transcripts were localized to the mitochondria, but there were significant differences in fluorescence intensity under identical conditions (Fig. 4A, C, and  E). Signal distribution analysis revealed that the fluorescence signals of BrSDH1-1A and BrSDH1-1C were much stronger than those of BrSDH1-1B (Fig. 4B, D, and  F). To quantitatively characterize these differences, we analyzed the expression of BrSDH1-1A, BrSDH1-1B, and BrSDH1-1C. The results confirmed that all three transcripts were successfully overexpressed and showed no significant differences at the transcriptional level (Fig. S4B). However, western blot analysis revealed that the protein expression level of BrSDH1-1A was considerably higher than that of BrSDH1-1B and BrSDH1-1C, which aligned with the subcellular localization data (Fig. S4C). These findings preliminarily suggested that *BrSDH1-1A*, *BrSDH1-1B*, and *BrSDH1-1C* may differ in expression abundance, and the underlying mechanism warrants further investigation. As *BrSDH1-1* is an emerging regulator of stomatal movement, studying the roles of these three transcripts is essential.

### OE-*BrSDH1-1A* and OE-*BrSDH1-1C* possessed more efficient drought tolerance and H_2_S responsiveness

**Figure 5:**
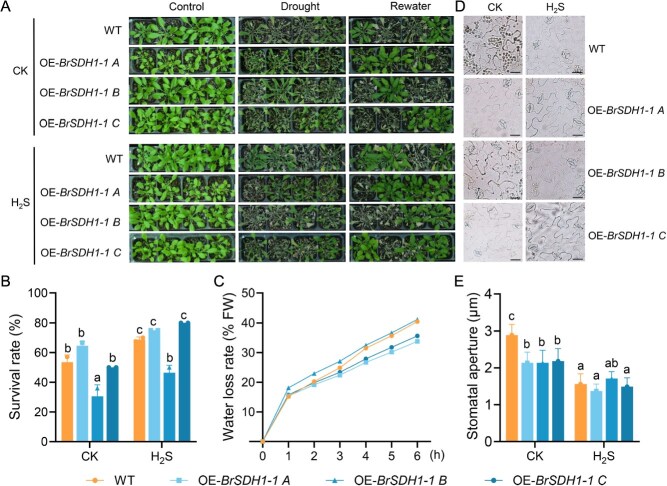
Overexpression of *BrSDH1-1* enhances drought tolerance. (A) Phenotype of *BrSDH1-1*-overexpressed lines and WT under natural drought for 7 days. (B, C) Survival rate and water loss rate were measured in WT, OE-*BrSDH1-1A*, OE-*BrSDH1-1B*, and OE-*BrSDH1-1C*. (D, E) Stomatal aperture of in WT, OE-*BrSDH1-1A*, OE-*BrSDH1-1B*, and OE-*BrSDH1-1C* were observed with the fumigation of H_2_S. Two-way ANOVA determined the significant differences (*P* < 0.05).

To more effectively explore the physiological function of *BrSDH1-1*, we overexpressed the three transcripts in *A. thaliana* (Fig. S5). Under normal growth conditions, no significant phenotypic differences were observed among the overexpression lines. However, after applying drought stress for 7 days through complete water deprivation, clear differences emerged (Fig. 5A). OE-*BrSDH1-1A* and OE-*BrSDH1-1C* plants remained greener and more turgid compared to OE-*BrSDH1-1B*, which exhibited severe wilting. Upon rewatering to soil saturation, seedling survival rates were quantified. Statistical results showed survival rates exceeding 50% in OE-*BrSDH1-1A* and OE-*BrSDH1-1C*, higher than the 30% observed in OE-*BrSDH1-1B* (Fig. 5B). It was evident that OE-*BrSDH1-1B* exhibited reduced drought resistance, as further indicated by water loss rate indices (Fig. 5C). To clarify the role of H_2_S in *BrSDH1-1* function, physiological concentrations of H_2_S were applied, which significantly enhanced survival rates (Fig. 5B). These results further confirmed that *BrSDH1-1* plays a crucial role in H_2_S-mediated drought resistance. As stomatal behavior is a vital indicator of drought tolerance, epidermal peels of WT, OE-*BrSDH1-1A*, OE-*BrSDH1-1B*, and OE-*BrSDH1-1C* were analyzed for stomatal aperture. As expected, *BrSDH1-1* overexpression promoted stomatal closure relative to WT, with no significant differences among OE-*BrSDH1-1A*, OE-*BrSDH1-1B*, and OE-*BrSDH1-1C* (Fig. 5D and E). H_2_S enhanced stomatal closure in WT, OE-*BrSDH1-1A*, and OE-*BrSDH1-1C*, but its effect on OE-*BrSDH1-1B* was negligible (Fig. 5D and E). These results implied that H_2_S differentially regulates OE-*BrSDH1-1A*, OE-*BrSDH1-1B*, and OE-*BrSDH1-1C*.

### H_2_S scavenged SDH-generated ROS and triggered BrSDH1-1 persulfidation

**Figure 6:**
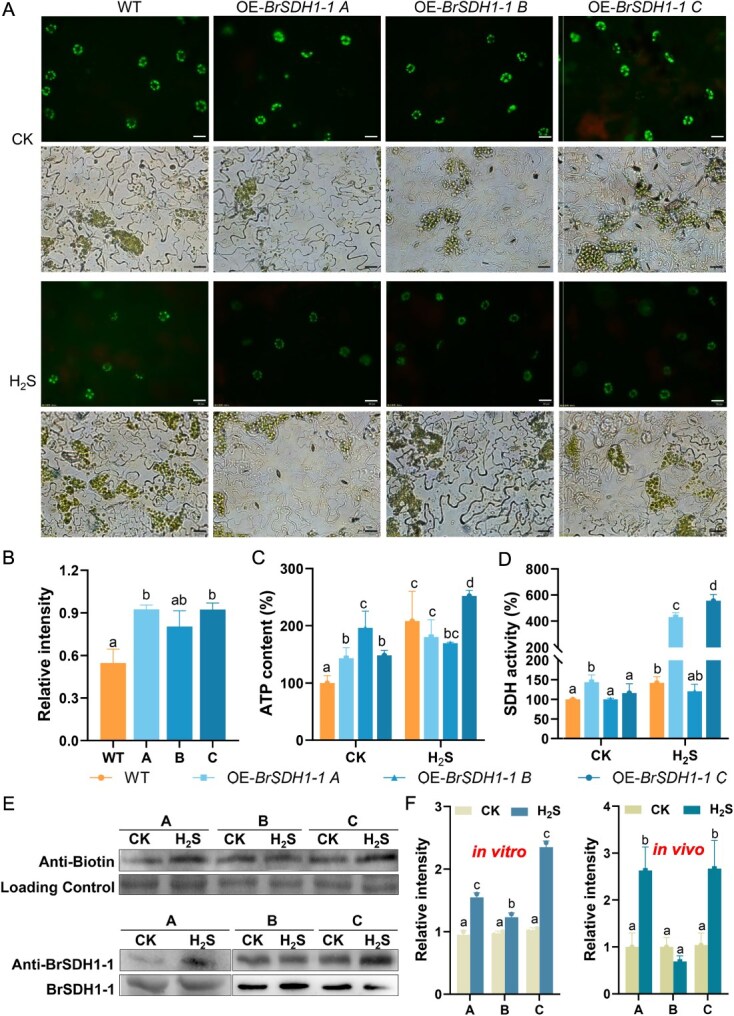
H_2_S persulfidated BrSDH1-1 to regulate ROS production. (A, B) ROS content in the GCs of WT, OE-*BrSDH1-1A*, OE-*BrSDH1-1B*, and OE-*BrSDH1-1C* were observed with the fumigation of H_2_S. Bar = 20 μm. (C) ATP content and (D) SDH activity were measured in WT, OE-*BrSDH1-1A*, OE-*BrSDH1-1B*, and OE-*BrSDH1-1C* with the fumigation of H_2_S. (E, F) Persulfidation level of BrSDH1-1 *in vitro* and *in vivo*. ‘A’, ‘B’, and ‘C’ indicate OE-*BrSDH1-1A*, OE-*BrSDH1-1B*, and OE-*BrSDH1-1C*, respectively*.* Two-way ANOVA determined the significant differences (*P* < 0.05).

Previous studies have demonstrated that SDH plays a critical role in regulating plant stress regulation [18, 19]. Our findings indicated that overexpression of *BrSDH1-1* enhanced ROS production, which was efficiently scavenged by H_2_S (Fig. 6A and B). However, the effect of H_2_S on OE-*BrSDH1-1B* was less evident compared to OE-*BrSDH1-1A* and OE-*BrSDH1-1C*, aligning with the observed patterns of stomatal closure (Fig. 6A and B). Since both ROS generation and stomatal behavior are closely linked to energy metabolism, we assessed ATP levels. The ATP content in OE-*BrSDH1-1A*, *B*, and *C* was clearly higher than in WT (Fig. 6C). Exogenous H_2_S application further stimulates ATP production in OE-*BrSDH1-1A* and OE-*BrSDH1-1*C, but not in OE-*BrSDH1-1B* (Fig. 6C). This observation was consistent with the drought tolerance shown by the OE-*BrSDH1-1* lines. To determine whether H_2_S promotes ATP production *via* BrSDH1-1, we measured SDH activity. The SDH activity in OE-*BrSDH1-1A* was significantly higher than in WT, while the increases in OE-*BrSDH1-1B* and OE-*BrSDH1-1C* were minimal (Fig. 6D). Upon treatment with physiological concentrations of H_2_S, SDH activity in OE-*BrSDH1-1A* and OE-*BrSDH1-1C* increased approximately four-fold compared to WT (Fig. 6D). However, H_2_S did not significantly enhance SDH activity in OE-*BrSDH1-1B*. The differential response of the OE-*BrSDH1-1* lines to H_2_S prompted us to investigate its mechanism of action on BrSDH1-1. Persulfidation assays revealed that BrSDH1-1A and BrSDH1-1C were strongly persulfidated by H_2_S, whereas BrSDH1-1B exhibited no clear persulfidation signals *in vitro* or *in vivo* (Fig. 6E and F). This may explain the inability of H_2_S to increase SDH activity and ATP production in OE-*BrSDH1-1B*. Based on these results, we propose that H_2_S modulates the SDH activity of OE-*BrSDH1-1A*, *B*, and *C* through persulfidation, thereby influencing ATP production and playing a role in BrSDH1-1-mediated stomatal movement and ROS signaling.

## Discussion

H_2_S has been recognized as a gasotransmitter for more than twenty years [33]. The rapid development of high-throughput sequencing technology offers the potential to comprehensively investigate the regulatory mechanisms of H_2_S. Proteomic analyses have established that H_2_S induces persulfidation of splicing factors—including CBP80, PTBP2, and PRP-19—which enhance plant stress resistance through coordinated modulation of multiple signaling pathways [22, 34]. AS is a common posttranscriptional regulatory mechanism in plants that enhances genetic information diversity, increasing organismal complexity and adaptability to environmental conditions [35]. Recent research has revealed that H_2_S participates in the AS of flowering-related genes by regulating the persulfidation of the splicing factor AtU2AF65a [32]. This finding provides insights into novel mechanistic research on H_2_S. Specifically, our study also reveals that the AS patterns of *BrSDH1-1* was modulated by H_2_S, generating functionally distinct isoforms under stress conditions (Figs 3–6). Collectively, these findings suggest that AS may represent another novel regulatory mechanism of H_2_S. However, the precise molecular mechanism underlying H_2_S-mediated regulation—specifically which splicing factors are targeted or at which regulatory node they act—remains uncharacterized. Given the nascent stage of research on H_2_S-mediated AS, future studies should prioritize identifying whether its regulatory scope involves modulation of spliceosomal components.

AS generates new transcripts and serves as an important regulatory mechanism in plants. Early investigations revealed that SDH1-1B and the ribosomal protein RPS14 are co-transcribed from a single pre-mRNA, yielding functionally distinct proteins through AS in *Oryza sativa* [36]. This finding establishes the first paradigm for investigating functional regulation of mitochondrial proteins through AS. Our results also indicated that H_2_S regulates the AS of *BrSDH1-1*, generating three transcript isoforms (Fig. 3). Accumulating evidence indicates that target protein abundance is frequently modulated by AS, enabling homeostatic growth under stress with minimal energy expenditure [37]. Research in *A. thaliana* has shown that PP2C-*HAB1* undergoes AS to toggle ABA signaling on and off, aiding plant adaptation to abiotic stress [38]. A study in *B. rapa* revealed that SR45a regulates drought tolerance by influencing the AS of target genes [39]. Our study further demonstrates that the *BrSDH1-1* splice variants exhibit significantly differing transcript abundances despite similar subcellular localization (Fig. 4). This regulatory modality, modulating isoform abundance of target proteins *via* AS, exhibits strong associations with translational efficiency and protein stability. Existing studies have shown that introducing an optimized Kozak sequence within a promoter construct improves the translation efficiency of downstream genes [40]. Additionally, ubiquitin-mediated degradation also affects protein abundance, as many AS transcripts may rapidly enter the ubiquitination pathway, resulting in functional differences [41].

Due to the small sequence differences, it was not possible to directly quantify changes in *BrSDH1-1A*, *BrSDH1-1B*, and *BrSDH1-1C* following H_2_S treatment. Therefore, we investigated their functional differences by overexpressing them separately in *A. thaliana*. Our results demonstrate that OE-*BrSDH1-1A* and OE-*BrSDH1-1C* exhibit significantly enhanced functional performance compared to OE-*BrSDH1-1B*, characterized by superior drought tolerance and a more pronounced response to H_2_S (Fig. 5). The known structural domains and subcellular localization of BrSDH1-1B was indistinguishable from that of BrSDH1-1A and BrSDH1-1C. However, BrSDH1-1B exhibits a loss-of-function phenotype, which may be linked to its translational efficiency (Fig. 4 and Fig. S4). The underlying mechanisms responsible for this functional difference therefore warrant further investigation. Abiotic stress leads to the production of ROS, and SDH is recognized as a major source of mitochondrial ROS [19]. Our findings also indicated that overexpression of *BrSDH1-1* promoted ROS accumulation, while excess ROS were scavenged by H_2_S to maintain ROS homeostasis (Fig. 6A and B). Analyses in animals have shown that H_2_S donates electrons *via* SQR and SDH to modulate immune responses through bioenergetic pathways [42]. In parallel, we observed that H_2_S enhanced SDH activity and ATP production, except in OE-*SDH1-1B* (Fig. 6C and D). Increased SDH activity has been shown to induce substantial accumulation of oxidized succinate in mitochondria, which in turn contributes to elevated ROS levels [43]. However, our results indicate that H_2_S scavenges ROS while concomitantly enhancing SDH activity, suggesting a sophisticated regulatory mechanism for maintaining organismal ROS homeostasis. ROS functions in a concentration-dependent manner, playing critical roles in signal transduction at low levels, while excessive ROS can cause oxidative damage to proteins and enzymes. Stomata can perceive a range of external and internal signals, and ROS act as key second messengers in regulating stomatal movement. Recent studies have demonstrated that mitochondrial ROS production triggers stomatal closure [44]. In relation to the phytohormone ABA, it has been shown that ABA increases mitochondrial H_2_O_2_ levels to promote stomatal closure [45]. Meanwhile, numerous oxidoreductases in mitochondria help monitor ROS levels to ensure they remain within a normal range. Studies in rats have established that ROS derived from SDH originate exclusively from the flavin site, contingent upon substrate occupancy at the oxidation site and the reduced state of SDH [46]. Subsequent research in *A. thaliana* revealed that both SDH point mutations and exogenous application of competitive inhibitors reduce ROS production, whereas non-competitive inhibitors markedly enhance ROS generation [18, 19]. And our data show that H_2_S both enhances SDH activity and scavenges excess ROS to sustain cellular redox homeostasis (Fig. 6). Collectively, these findings provide a mechanistic framework for identifying the precise site(s) of H_2_S action on SDH and reveal that mitochondria function not merely as cellular powerhouses, but as signaling hubs that actively modulate complex stress responses in plants.

Since *BrSDH1-1B* did not respond to H_2_S, the functional differences among *BrSDH1-1A*, *BrSDH1-1B*, and *BrSDH1-1C* became more pronounced following H_2_S application (Fig. 6A–D). Variations in the persulfidation levels of BrSDH1-1A, BrSDH1-1B, and BrSDH1-1C, both *in vitro* and *in vivo*, provide molecular evidence for their differing responses to H_2_S (Fig. 6E and F). While H_2_S typically induces persulfidation at cysteine residues, the impaired modification of BrSDH1-1B implies sequence-dependent conformational changes that limit H_2_S binding. Definitive verification necessitates resolving splice variant structures alongside functional proteomics to identify persulfidation sites. The combined advancement of high-throughput sequencing and multi-omics technologies now allows for accurate quantification of transcript changes, offering a more robust foundation for multi-dimensional screening of genes with strong stress resistance and desirable preservation traits.

As an important economic crop, the growth and productivity of Chinese cabbage are increasingly threatened by adverse abiotic stresses [5]. We successfully silenced *BrSDH1-1* using VIGS, demonstrating its involvement in stomatal movement in Chinese cabbage, a process closely linked to energy metabolism (Fig. 1). However, genetic transformation is significantly constrained by the low regeneration frequency of Chinese cabbage. Therefore, we validated the function of different splice variants of *BrSDH1-1* in *A. thaliana*, and the development of a robust tissue culture system remains essential. Functional validation through heterologous expression of *B. rapa* genes in the model plant *A. thaliana* is a widely utilized strategy [47]. In addition, the rapid advancement of high-throughput transcriptome sequencing technologies is expected to significantly impact future horticultural research [48].

Notably, far more genes were induced by H_2_S than by HA, likely because HA only inhibits the activity of D-cysteine desulfhydrase (Fig. 2A–D). It is well known that H_2_S production in plants primarily occurs *via* cysteine metabolism. In addition to D-cysteine desulfhydrase, L-cysteine desulfhydrase (EC 4.4.1.1), O-acetyl-L-serine (thiol) lyase (OAS-TL) (EC 4.2.99.8), 3-mercaptopyruvate sulfurtransferase (EC 2.8.1.2), and NFS all contribute to H_2_S [33]. Therefore, it is expected that only a limited number of genes were differentially expressed following HA treatment. Our RNA-Seq data similarly confirmed that mitochondria are major responders to H_2_S (Fig. 2F). However, given the inherent limitations of RNA-Seq, these data only provide preliminary support for a positive response of mitochondrial proteins to H_2_S. To robustly validate this proposed mechanism, targeted experiments focused on assessing the direct impact of H_2_S on mitochondrial proteins are required. Direct studies on mitochondria have shown that H_2_S delays leaf senescence by preserving mitochondrial integrity [49]. Investigations of individual mitochondrial complexes have revealed that H_2_S influences stomatal movement *via* cytochrome *c* [50]. Previous research demonstrated that H_2_S induces stomatal closure through persulfidation of SDH1-1 in *A. thaliana* [30]. Consistent with this, the current study found that H_2_S-regulated stomatal movement partially depends on *BrSDH1-1*, though its role in promoting ATP production remained evident even in *BrSDH1-1-*silenced lines (Fig. 1C–F). This may be because plant cells compensate by utilizing Complex I for electron transfer when Complex II is impaired, maintaining normal growth, development, and stress responses. This observation suggests that the mitochondrial targets of H_2_S may not be limited to a single complex, further highlighting the necessary to investigate other components of the ETC to fully elucidate H_2_S target molecules. Interestingly, H_2_S did not affect *BrSDH1-1* expression at the transcriptional level (Fig. 2G). However, it did influence the AS of *BrSDH1-1*, providing a foundation for exploring the posttranscriptional regulatory mechanisms of H_2_S (Fig. 3A and B).

**Figure 7:**
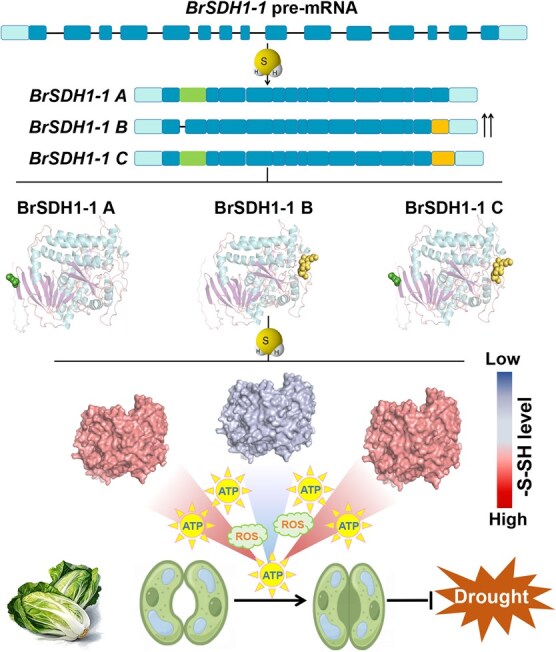
Proposed model of H_2_S modulates the AS and persulfidation of BrSDH1-1 to affect ROS production, ATP generation, and drought tolerance in Chinese cabbage. Gene model of the different BrSDH1-1 isoforms generated by AS is displayed. Triangular shapes represent ATP production and the darker the color, the more signal accumulates. Pre-mRNA, precursor messenger RNA; H_2_S, hydrogen sulfide; ROS, reactive oxygen species.

Taken together, we elucidate that H_2_S induces the AS of *BrSDH1-1*, generating three transcripts that differentially contribute to H_2_S-mediated energy biosynthesis and stomatal closure, alongside ROS scavenging in Chinese cabbage (Fig. 7). This indicates that *BrSDH1-1* may serve as a potential target through which H_2_S regulates the abiotic stress response *via* energy metabolism—an insight with broad agricultural significance. More importantly, we provide evidence supporting AS as a novel molecular mechanism underlying the regulatory functions of H_2_S.

## Materials and methods

### Plant material and growth conditions

Heading Chinese cabbage seeds ‘Aiqing 1#’ (*B. rapa*) were provided by Prof. Jiashu Cao (Zhejiang University, China). Heading Chinese cabbage, *Nicotiana benthamiana* and *A. thaliana* (Col-0) seeds were grown in a greenhouse under long-day conditions (16 h light/8 h dark at 23°C ± 1°C).

To investigate the wide-ranging effects of H_2_S on gene expression in Chinese cabbage, the seeds were dark-cultured for 24 h in a dish at 4°C and then transplanted to the greenhouse. A 50-μM NaHS (H_2_S donor) solution and 1-mM hydroxylamine (HA, an H_2_S inhibitor) were used to spray 10-day-old seedlings for 4 h. Equal amounts of water were used as a control. Seedlings were harvested for RNA-Seq.

To analyze the effect of H_2_S on BrSDH1-1 in Chinese cabbage, control plants (pTY-S) and pTY-*BrSDH1-1*, over-expression of different transcripts of *BrSDH1-1* in *A. thaliana* and wild type (WT) were treated with 50 μM NaHS.

To study the response of BrSDH1-1 to drought stress, overexpressing *BrSDH1-1* in *Arabidopsis* and WT plants were subjected to natural drought for 7 days.

### Virus-induced gene silencing

The pTY-S vector was provided by Prof. Ying Li (Nanjing Agricultural University, China). A 40-bp fragment of *BrSDH1-1* was designed and reverse-complemented to form an 80-bp palindrome structure using GenScript (Nanjing, China; target sequences are listed in Fig. S2). pTY-S vector was used as a negative control. Two-week-old Chinese cabbage seedlings were used for the gene silencing assay [51]. To get a favorable bombardment efficiency, the vacuum level in bombardment chamber is 28 Hg, helium pressure is set at 1350 psi, the target distance is 9 cm, and 0.6 μm gold is used as the microarrier. Plants treated with the gene gun were left in the dark for 12 h and then transferred to the greenhouse. After 21 days, total RNA from plants showing mosaic symptoms was extracted to analyze silencing efficiency (for primer sequences used for silencing validation, see Table S2), and the silenced plants were used for further assays.

### Extraction of total RNA and RT-PCR

Total RNA was extracted from leaf tissues using TRIzol™ Reagent (Takara, Kyoto, Japan). Reverse transcription was performed with the 5× All-In-One RT Master Mix (Applied Biological Materials, Vancouver, Canada) according to the manufacturer’s protocol. Semi-quantitative RT-PCR was carried out to determine the expression level of target genes using *BrACTIN* as an internal control. PCR products were separated on 1% agarose gels, stained with GelRed™ Nucleic Acid Gel Stain (Yeasen Biotechnology, Shanghai, China), and quantified by densitometry using ImageJ. Target gene expression levels were normalized to *BrACTIN* intensity ratios.

### RNA-Seq data and qRT-PCR analysis

Approximately 1 μg of total RNA from 10-day-old Chinese cabbage seedlings, fumigated with 50 μM NaHS or 1 mM HA, was prepared for cDNA libraries. Three independent biological repeats were conducted for each treatment. Gene expression was determined using the previously described protocol [30]. The gene-specific primers for qPCR are listed in Table S2, and *BrACTIN* was used as the internal standard, with three independent biological replicates set up.

### Analysis of differentially expressed genes and AS events

To identify differentially expressed genes (DEGs) after treatment with H_2_S and HA, the expression levels of genes from transcriptome data were statistically determined. The detailed method for DEG and AS event analysis was performed following the previous study [32].

### Stomatal aperture assays

Stomatal aperture measurement was conducted as per our previous study [30]. To investigate the role of BrSDH1-1 in stomatal movement, epidermal strips were collected from Chinese cabbage leaves subjected to gene gun treatment. The epidermal peels of pTY-S plants and pTY-*BrSDH1-1* plants were divided into three groups: one group was directly fixed and used to observe stomatal aperture size, while the other two were floated in buffer containing 10 mM 2-(*N*-morpholino) ethanesulfonic acid (MES) and 50 mM KCl under light for 2 h. These strips were then treated with or without 50 μM NaHS for 1 h to observe differences in stomatal movement. Rosette leaves from 4-week-old overexpressed-*BrSDH1-1 Arabidopsis* were used for the stomatal aperture assay, following the same treatment as for Chinese cabbage.

### Water loss rate

About ten mature leaves from WT, OE-*BrSDH1-1A*, OE-*BrSDH1-1B*, and OE-*BrSDH1-1C* were sampled, and the fresh weight was recorded as W0. Each sample was then dried at room temperature for 6 h until the fresh weight stabilized. All leaves were weighed every hour as W1. The ratio of water loss (RWL) was calculated as RWL = (W0 − W1)/W0 × 100%.

### Detection of ATP content

ATP content was measured according to the method previously described [30]. Leaves of pTY-S/pTY-*BrSDH1-1* plants and 4-week-old *A. thaliana* seedlings were used.

### Detection of ROS content

Lower epidermal strips were peeled from leaves of selected 4-week-old WT, OE-*BrSDH1-1A*, OE-*BrSDH1-1B*, and OE-*BrSDH1-1C.* Then the tissues were incubated with or without H_2_S in buffer (50 mM KCl, 10 mM MES-KOH, pH 6.15) containing 10 μM dichlorofluorescin diacetate (DCFH-DA, Sigma), a ROS fluorescent probe for 30 min. After dye incubation, the tissues were washed with buffer (50 mM KCl, 10 mM MES-KOH, pH 6.15) for three times. ROS signal distribution indicated by green fluorescent signal was acquired at excitation of 488 nm and collected in 515–560 nm wavelength range using the confocal microscope system (Olympus Corporation, Japan).

### Subcellular localization analysis

Coding sequences of *BrSDH1-1A*, *BrSDH1-1B*, and *BrSDH1-1C* were amplified and independently cloned into the p1305-GFP vector to perform subcellular localization assays. The constructed plasmids were transformed into *Agrobacterium tumefaciens* (EHA105) and injected into 3-week-old *N. benthamiana*. The fluorescence signal was visualized by confocal microscopy; excitation and emission wavelengths for GFP were 488/493 to 598, and for RFP were 561/595 to 670 (ZEISS, Germany).

### Immunochemical detection of persulfidated BrSDH1-1

Persulfidated protein levels were detected *in vitro* according to our previous methods [29]. Briefly, purified recombinant proteins were treated with 1 mM NaHS at 4°C for 30 min. The persulfidated proteins were detected by immunoblotting using a biotin antibody (anti-biotin, Abcam, Cambridge, UK; 1:5000). *In vivo* levels of persulfidated BrSDH1-1 were detected in overexpressing plants. Ten-day-old 35S::*BrSDH1-1A*, 35S::*BrSDH1-1B*, and 35S::*BrSDH1-1C* transgenic seedlings were treated with 200 μM NaHS for 40 min. The seedlings were then ground with liquid nitrogen and homogenized in extraction buffer (25 mM Tris, 100 mM NaCl, 0.2% (v/v) Triton X-100, pH 8.0). Persulfidated proteins were detected using an SDH1-1 antibody at a dilution of 1:4000 (PhytoAB, San Francisco, CA, USA).

### SDH enzyme activity assay

The detection of SDH enzyme activity was previously described in detail [30]. Briefly, pTY-S plants, pTY-*BrSDH1-1* plants, and *BrSDH1-1*-overexpressed *Arabidopsis* were fumigated with or without 50 μM NaHS for 10 min to assess SDH enzyme activity. The CheKine™ Mitochondrial Complex II Activity Assay Kit (Sigma-Aldrich, St. Louis, MO, USA) was used to determine SDH activity levels according to the manufacturer’s instructions.

### Statistical analyses

Three biological and three technical replicates were performed for each experiment, and the mean ± SD represents the summary statistics of the data. One-way ANOVA and two-way ANOVA using SPSS (version 22, IBM SPSS, Chicago, IL, USA) were employed for data analysis, with *P <* 0.05 as the threshold. Significant differences were indicated using different lowercase letters.

## Supplementary Material

Web_Material_uhaf214

## Data Availability

All data can be found in the main text and supplemental information.
