# Supplementary material for: H2S modulates BrSDH1-1 alternative splicing to induce stomatal closure in Chinese cabbage
Source: Hortic Res. 2025 Aug 22;12(11):uhaf214. doi: 10.1093/hr/uhaf214 (PMC12580989; doi:10.1093/hr/uhaf214)
Supplement: Web_Material_uhaf214 [file web_material_uhaf214.zip › Supplementary Materials-new.docx]

**Supplementary material**

**Supplementary Figure 1**


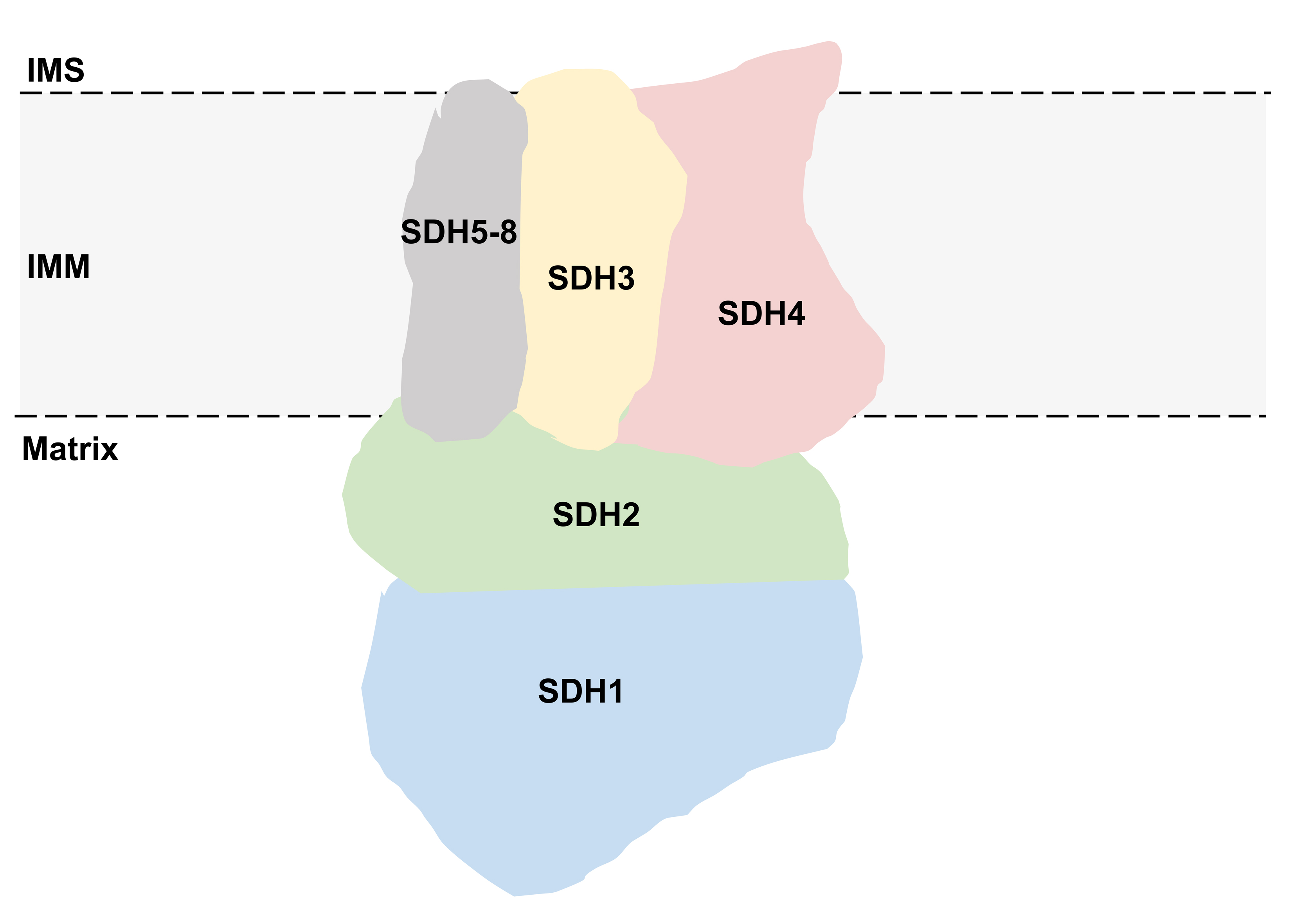


**Fig. S1.** The structure of SDH. IMS, intermembrane space; IMM, inner mitochondrial membrane.

**Supplementary Figure 2**


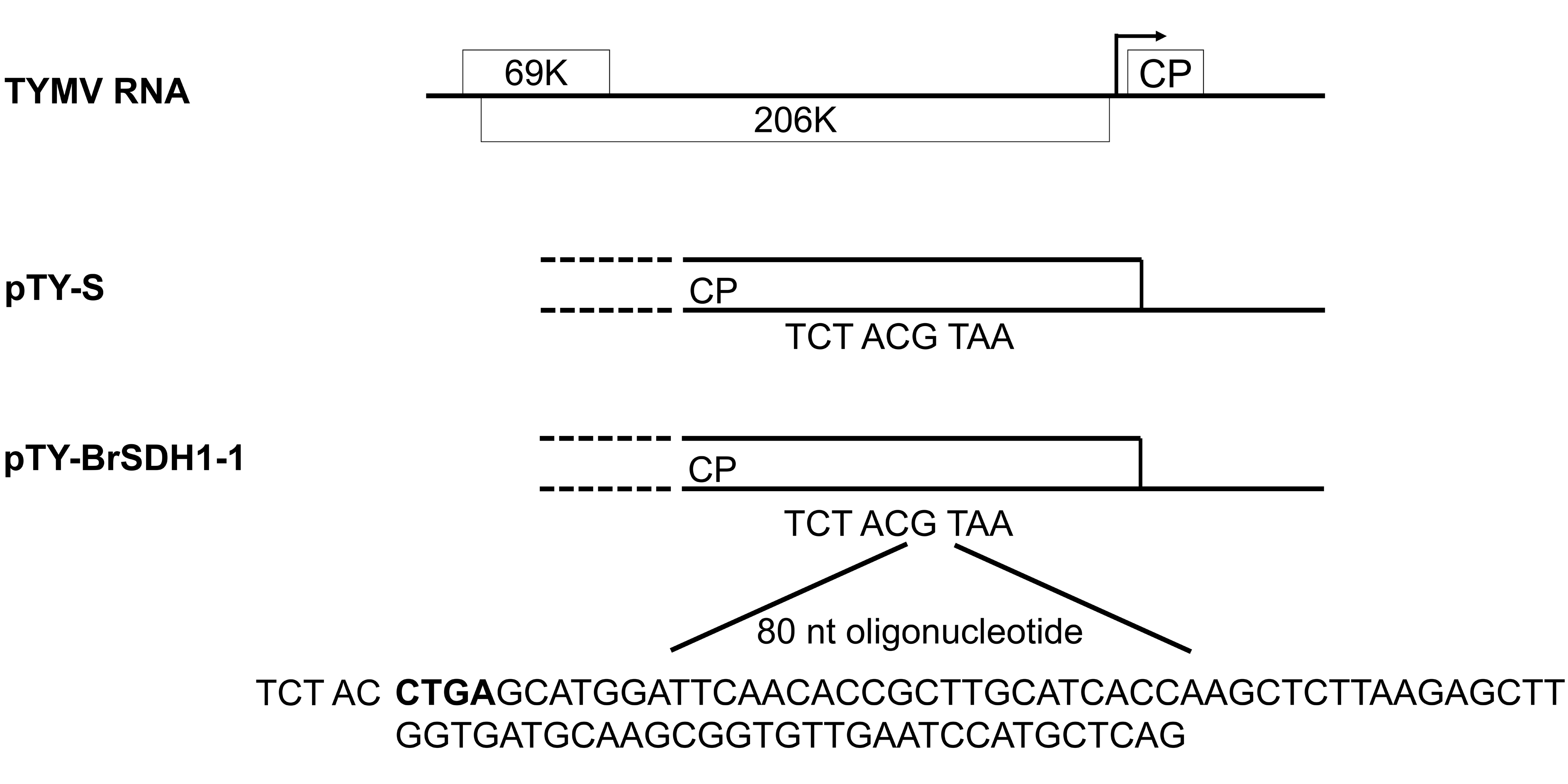


**Fig. S2.** Genomic organization of pTY-S and details for vector construction.

**Supplementary Figure 3**


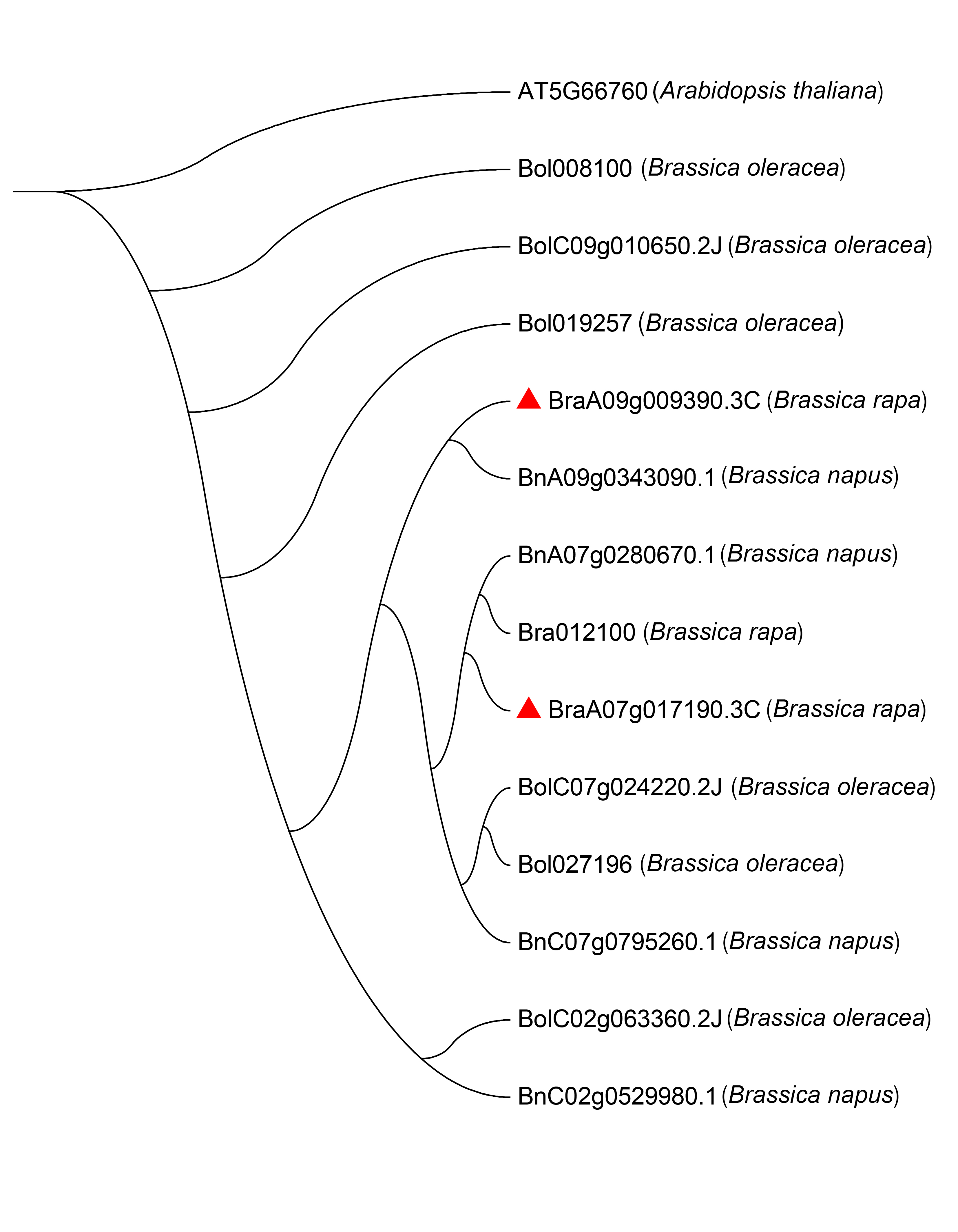


**Fig. S3.** Phylogenetic tree analysis of *BrSDH1-1*, its homologs and orthologs from various plants. Red triangles represent gene IDs corresponding to *BrSDH1-1*.

**Supplementary Figure 4**


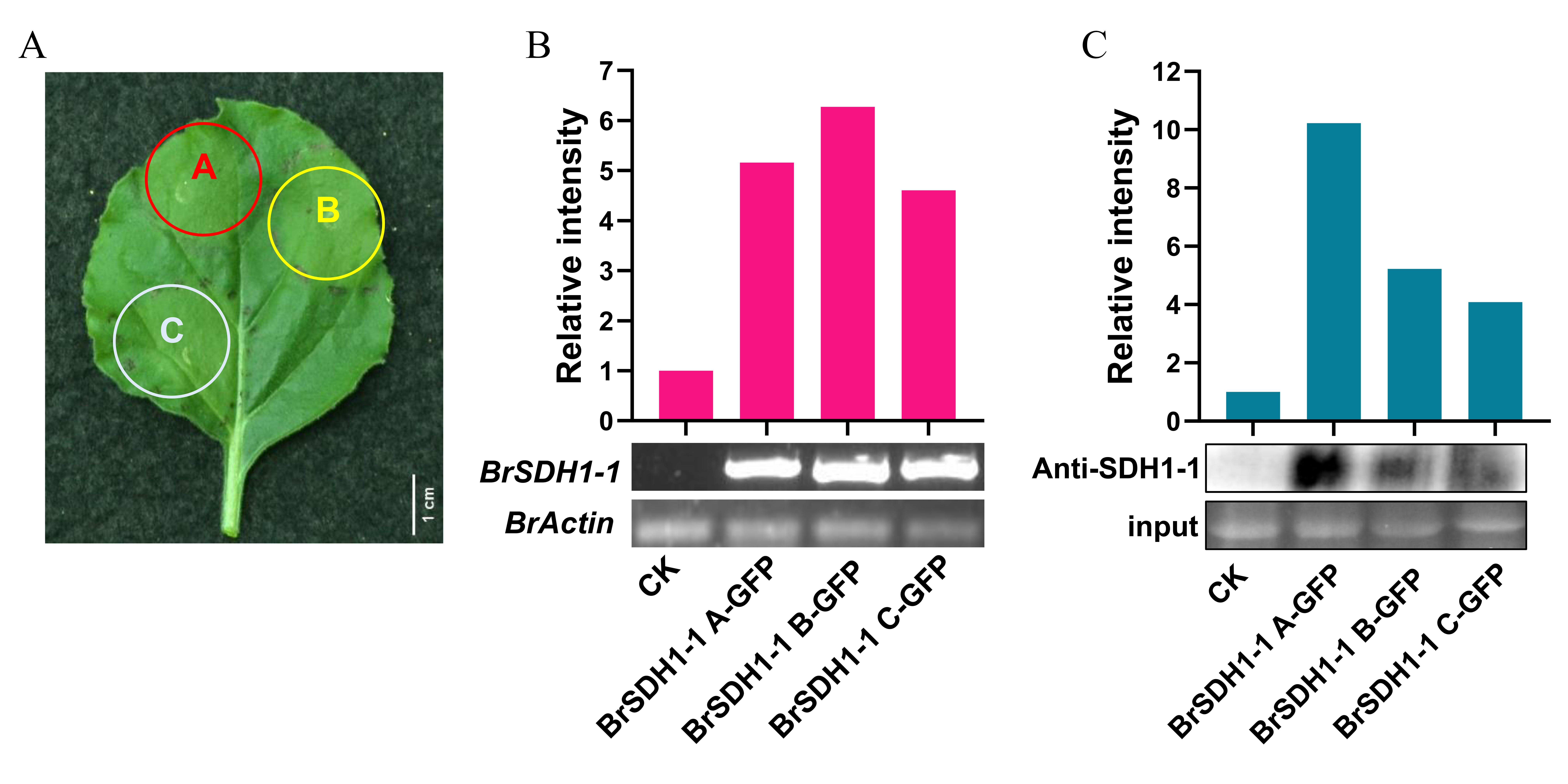


**Fig. S4.** The expression of *BrSDH1-1* in tobacco leaves at the transcriptional and protein translation level. **A** Schematic diagram of quantitative injection of BrSDH1-1 A-GFP, BrSDH1-1 B-GFP and BrSDH1-1 C-GFP. “A”, “B”, and “C” stands for BrSDH1-1 A-GFP, BrSDH1-1 B-GFP and BrSDH1-1 C-GFP respectively. The expression of BrSDH1-1 at the transcriptional level (**B**) and protein translation level (**C**) in tobacco leaves after injection of BrSDH1-1 A-GFP, BrSDH1-1 B-GFP and BrSDH1-1 C-GFP.

**Supplementary Figure 5**

**
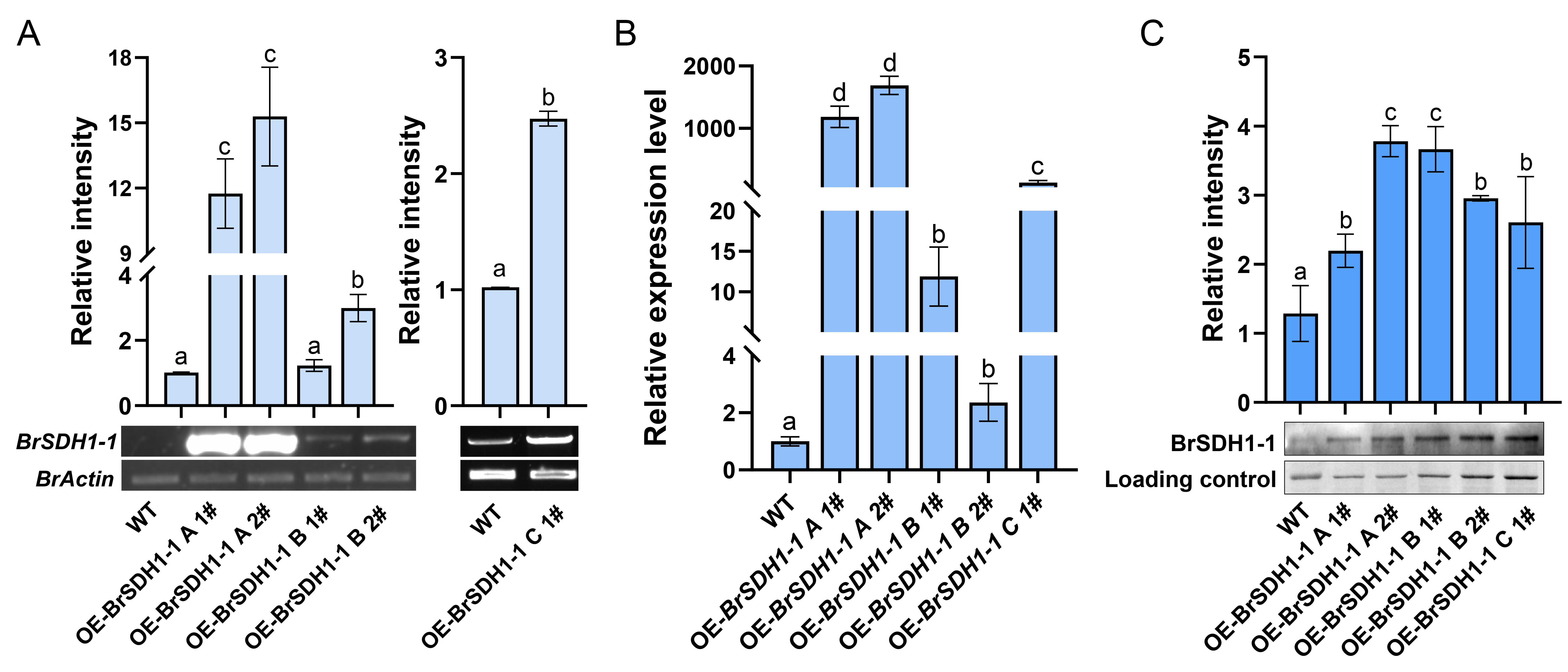
**

**Fig. S5.** The analysis of gene expression in OE-*BrSDH1-1A*, OE-*BrSDH1-1B* and OE-*BrSDH1-1C* plants. Expression analysis of *BrSDH1-1* as assessed by (A) RT-PCR, (B) qRT-PCR, and (C) Western blot. One-way ANOVA determined the significant differences (*p* < 0.05).
